# Nucleotide heterogeneity at the terminal ends of the genomes of two California *Citrus tristeza virus* strains and their complete genome sequence analysis

**DOI:** 10.1186/s12985-018-1041-4

**Published:** 2018-09-15

**Authors:** Angel Y. S. Chen, Shizu Watanabe, Raymond Yokomi, James C. K. Ng

**Affiliations:** 1 Department of Microbiology and Plant Pathology, University of California, Riverside, CA 92521 USA; 2 Center for Infectious Diseases and Vector Research, University of California, Riverside, CA 92521 USA; 3 United States Department of Agriculture, Agricultural Research Service, Parlier, CA 93648 USA

**Keywords:** Citrus tristeza virus, Genotype, RACE, Heterogeneity, Genome ends

## Abstract

**Background:**

The non-translated regions at the genome ends of RNA viruses serve diverse functions and can exhibit various levels of nucleotide (nt) heterogeneity. However, the extent of nt heterogeneity at the extreme termini of *Citrus tristeza virus* (CTV) genomes has not been comprehensively documented. This study aimed to characterize two widely prevalent CTV genotypes, T36-CA and T30-CA, from California that have not been sequenced or analyzed substantially. The information obtained will be used in our ongoing effort to construct the infectious complementary (c) DNA clones of these viruses.

**Methods:**

The terminal nts of the viral genomes were identified by sequencing cDNA clones of the plus- and/or minus-strand of the viral double-stranded (ds) RNAs generated using 5′ and 3′ rapid amplification of cDNA ends. Cloned cDNAs corresponding to the complete genome sequences of both viruses were generated using reverse transcription-polymerase chain reactions, sequenced, and subjected to phylogenetic analysis.

**Results:**

Among the predominant terminal nts identified, some were identical to the consensus sequences in GenBank, while others were different or unique. Remarkably, one of the predominant 5′ nt variants of T36-CA contained the consensus nts “AATTTCAAA” in which a highly conserved cytidylate, seen in all other full-length T36 sequences, was absent. As expected, but never systematically verified before, unique variants with additional nt (s) incorporated upstream of the 5′ terminal consensus nts of T36-CA and T30-CA were also identified. In contrast to the extreme 5′ terminal nts, those at the extreme 3′ termini of T36-CA and T30-CA were more conserved compared to the reference sequences, although nt variants were also found. Notably, an additional thymidylate at the extreme 3′ end was identified in many T36-CA sequences. Finally, based on pairwise comparisons and phylogenetic analysis with multiple reference sequences, the complete sequences of both viruses were found to be highly conserved with those of the respective genotypes.

**Conclusions:**

The extreme terminal nts in the T36-CA and T30-CA genomes were identified, revealing new insights on the heterogeneity of these CTV genomic regions. T36-CA and T30-CA were the first and the second genotypes, respectively, of CTV originating from California to be completely sequenced and analyzed.

**Electronic supplementary material:**

The online version of this article (10.1186/s12985-018-1041-4) contains supplementary material, which is available to authorized users.

## Background

The family *Closteroviridae*, to which *Citrus tristeza virus* [CTV] belongs, comprises members with single-stranded (ss)-, positive polarity RNA genomes that are the largest among plant-infecting viruses. The approximately 19.3 k bases (kb) genome of CTV exists as a monopartite RNA component and consists of 12 genes potentially encoding 19 protein products [1, 2]. Based on the analysis of complete genome sequences using a > 7.5% average nt variation as a guide for delineating genotypes, at least six major genotypes, T36, T30, RB, VT, T3, and T68, exist among CTV strains [3, 4]. Among the different CTV genotypes, the frequency of variations at the genomic 5′ half is generally higher than that of the 3′ half [5, 6]. Within each genotype, as with all RNA viruses, CTV isolates exist as a population with one predominant consensus sequence accompanied by a pool of genetic variants called quasispecies [7–10]. The 5′ and 3′ terminal regions in the genomes of RNA viruses play important roles in many biological processes, such as translation, replication, virion assembly and pathogenesis [11–16]. Therefore, a detailed knowledge of the nt heterogeneity at the ends of the CTV genome would contribute an indispensable perspective to our understanding of CTV functions and applications.

The current work is an important prelude to the construction of infectious cDNA clones of California (CA) strains of CTV to be used as a reverse genetics platform for studying viral gene functions, and for molecular biotechnology applications that are specific to the needs of California. Specifically, knowledge of the nt variations within each CTV genotype would guide the design of molecular cloning strategies aimed at incorporating these nts into the infectious cDNA clones. To this end, we have focused on determining the heterogeneity at the extreme termini of the genomes of two prevalent strains of CTV with the T36 and T30 genotypes (from here on referred to as T36-CA and T30-CA, respectively) that are widely distributed throughout California. Symptoms associated with T30-CA and T36-CA infection vary on different citrus hosts and may include leaf cupping, vein clearing, stem pitting, seedling yellow, and quick decline. In citrus scions that are grafted on most commercially grown CTV tolerant rootstocks, T30-CA and T36-CA are associated with relatively mild to sometimes asymptomatic infection. While data on polymorphism within the 5′ and 3′ untranslated regions (UTR) s of the CTV genome exist [5, 17, 18], there is no published information on the stringent and comprehensive assessments of the heterogeneity at the extreme ends of CTV genomes in the literature. Furthermore, while the complete sequence of CTV with the T30 genotype isolated from *Citrus reticulata* Blanco (Murcott mandarin) in Fillmore, CA became available recently [4], whole genome sequence information of other isolates with the T30 genotype from California as well as CTV with the T36-CA genotype have not been determined. Thus, knowledge of the heterogeneity of the genomes and genome ends of California CTV strains exhibiting these two genotypes remains limited.

In the first part of this study, we analyzed the nt heterogeneity at the extreme genome ends of T36-CA and T30-CA. This information facilitated the design of specific strategies to clone the predominant genomic sequences of T36-CA and T30-CA. In the second part of this study, the complete nt sequences of T36-CA and T30-CA were assembled and compared with those of reference sequences of the same or different genotypes as our two queried sequences. To our knowledge, T36-CA and T30-CA are the first T36 and the second T30 genotypes, respectively, originating from California to be completely sequenced and analyzed.

## Methods

### CTV sources and double stranded (ds) RNA extraction

Bark tissues of Madam Vinous plants infected with CTV isolate P109.1a (CCTEA 96339; with the T36 genotype [19]) originating from Ventura (in the city of Fillmore) and Tulare counties, CA were used for dsRNA purification by CF-11 cellulose column chromatography [20, 21]. An asymptomatic CTV isolate 702 5a (with the T30 genotype) obtained from Mexican lime trees, also from Fillmore, CA were collected for dsRNA extraction using either the double-RNA viral dsRNA extraction mini kit (iNtRON Biotechnology, Korea) according to the manufacturer’s instructions, or by CF-11 cellulose column chromatography. The use of CTV-infected plant materials was in compliance with the California Department of Food and Agriculture regulation on the movement and handling of plant pathogens (permit no. 3081).

### 5′ RNA ligase-mediated rapid amplification of cDNA ends (RLM-RACE)

The 5′ region of the plus (+)-strand of CTV dsRNA was amplified by RLM-RACE using the Gene Racer kit (Life Technologies, Carlsbad, CA) according to the manufacturer’s instructions with three modifications. First, CTV dsRNA was denatured with 20 mM methylmercury hydroxide [20] at room temperature for 10 min, and the reaction was quenched with 100 mM β-mercaptoethanol at room temperature for 5 min. Second, dephosphorylation by Calf intestinal phosphatase (CIP) treatment was excluded; the denatured dsRNA was directly treated with tobacco acid pyrophosphatase (TAP) to remove the 5′ cap structure and ligated to an RNA oligo (provided in the Gene Racer kit). Third, the Herculase II fusion DNA polymerase (Agilent Technologies, Santa Clara, CA) was used for all PCR reactions. All oligo primers used for 5′ RLM-RACE are listed in Additional file 1: Table S1. The first strand cDNA was generated by reverse transcription (RT) using SuperScript III reverse transcriptase (Life Technologies) and oligo primers CTV349–AC or CTV345-AC. The cDNA corresponding to the 5′ region of the (+)-RNA of T36-CA or T30-CA was PCR-amplified using the GeneRacer 5′ primer and oligo primer CTV349–AC or CTV345-AC. The 839 and 969 bp PCR products generated using the T36-CA and T30-CA templates, respectively, were subjected to nested PCR using the GeneRacer 5′ nested primer and primers CTV350-AC or CTV346–AC. The resulting products amplified using the T36-CA and T30-CA sequences were 573 bp and 560 bp, respectively.

### 3′ rapid amplification of cDNA-ends (RACE)

Purified dsRNA was heated at 94 °C for 5 min [10, 22] and subjected to 3′ RACE using the 3′ RACE system for rapid amplification of cDNA ends kit (Life Technologies) according to the manufacturer’s instructions with some modifications [23]. The (+)- and minus (−)-RNA were polyadenylated at their 3′ ends with *E. coli* poly (A) polymerase (New England Biolabs Inc. Ipswich, MA) according to the manufacturer’s instructions, and then reverse transcribed using SuperScript II reverse transcriptase and an oligo (dT) primer. The resulting cDNA was subjected to PCR and nested PCR using the CTV-specific oligo primers listed in Additional file 1: Table S1. The 3′ RACE-generated products that corresponded to the 5′ and 3′ regions of the T36-CA genome was approximately 472 bp and 444 bp, respectively, while the 3′ RACE-generated products that corresponded to the 5′ and 3′ region of the T30-CA genome was approximately 311 bp and 407 bp, respectively. All PCR amplifications were performed using the Herculase II fusion DNA polymerase.

### Cloning of RACE products and nucleotide sequence analysis

The DNA products generated by 5′ RLM-RACE and 3′ RACE were each resolved by electrophoresis in a 1% agarose gel and purified using the MinElute gel extraction kit (Qiagen, Valencia, CA). The gel-purified 5′ RLM-RACE- and 3′ RACE-derived DNA were cloned using the Zero blunt TOPO PCR cloning kit for sequencing (Life Technologies) and the pGem T-Easy vector (Promega, Madison, WI), respectively, according to the manufacturer’s instructions, and transformed into *E. coli* (DH5α). Multiple clones were selected and sequenced to determine the nt identities of the cloned DNA. DNA sequencing was conducted in the Genomics Core of the Institute for Integrative Genome Biology (IIGB) at the University of California, Riverside. Selected CTV sequences with the T30 genotype (GenBank accession nos. KC517489, KC517490, KC517491, AF260651, Y18420, KC748391 and KU578007) and T36 genotype (GenBank accession nos. KC517485, KC517487, EU937521 and AY170468) were used for nucleotides comparison using the Clustal Omega program (https://www.ebi.ac.uk/). The identities of the most commonly seen nt variants as well as the frequency with which these variant nts appeared in the cloned DNA are shown in Tables 1 to 2.

### Generating the cDNA clones of CTV genomes

The methylmercury hydroxide denatured dsRNA of T36-CA or T30-CA described above was reverse transcribed to generate the first strand cDNA. The RT reaction was performed using the Maxima H minus reverse transcriptase (Thermo Scientific) and the oligo primer CTV30-AC according to the manufacturer’s instructions. CTV30-AC and other oligo primers used for PCR amplification are listed in Additional file 1: Table S1. The 5′ fragment (nt 1 to 8391) of T36-CA was amplified by PCR using the oligo primers CTV34-AC and CTV39-AC, and the 3′ fragment (nt 7800 to 19,292) was PCR-amplified using oligo primers CTV38-AC and CTV35-AC. The 5′ fragment (nt 1 to 8430) of T30-CA was amplified by PCR using the CTV32-AC and CTV37-AC primers, and the 3′ fragment (nt 7584 to 19,259) was PCR-amplified using CTV36-AC and CTV33-AC primers. The Expand 20 Kb ^plus^ PCR system (Roche) was used for the PCR-amplification. The RT-PCR products were gel-purified, cloned into pGem T Easy (Promega) and transformed into *E. coli*, JM109 (Promega), and several cDNA clones of each fragment were sequenced as described above. The nt sequences of full-length CTV genomes were assembled using the Vector NTI software (Life Technologies), and the complete sequences of T36-CA and T30-CA were deposited in the NCBI GenBank database with accession nos. MH279617 and MH279618, respectively.

### Sequence alignment and phylogenetic analysis

Nucleotide and deduced amino acid (aa) sequences were aligned using the MUSCLE and Clustal Omega programs, respectively, available on the website: https://www.ebi.ac.uk/, to determine the nt and aa identities among CTV isolates. The 2D color-coded matrix of pairwise nucleotide identity was generated using the Sequence Demarcation Tool v1.2 [24]. The CTV isolates selected for pairwise comparisons of nt and aa sequences were obtained from the NCBI GenBank database and included T36-FL (GenBank accession no. AY170468) [13], T30-FL (GenBank accession no. AF260651) [5], T30-AT4 (GenBank accession no. KU578007) [4], SY568 (GenBank accession no. AF001623) [25], RB-AT25 (GenBank accession no. KU356007) [26] and VT-AT39 (GenBank accession no. KU361339) [4].

Phylogenetic analysis was performed using MEGA 7 [27]. A phylogenetic tree was constructed using the complete genome sequences of T36-CA and T30-CA, as well as those of CTV isolates with the six major genotypes, T36, T30, RB, VT, T3, and T68 [3]. The specific names and accession numbers of all isolates used in the analysis are indicated in Fig. 2. CTV phylogeny was inferred using the maximum parsimony (MP) method, and the tree was generated using the Subtree-Pruning-Regrafting (SPR) algorithm with search level 1 in which the initial trees were obtained by the random addition of sequences (10 replicates) [28]. The reliability of the tree was estimated using the bootstrap test with 1000 replicates [29].

## Results

### Nucleotide heterogeneity at the genome ends of T36-CA

A comparison of multiple T36 full-length genomic sequences from GenBank indicated that the nt sequences at the extreme 5′ end may be placed in two consensus groups. The group 1 consensus sequence (e.g. GenBank accession nos. KC517485 and KC517487) is “AATTTCTCAA” (readers should note that we have followed GenBank’s nt annotation format by using “T” (thymidylate), rather than “U” (uridylate), in all the nt sequences determined by DNA sequencing). The group 2 consensus sequence (e.g. GenBank accession nos. EU937521 and AY170468) is “AATTTCACAA”. Analysis of 195 (+)- and (−)-DNA clones derived from the (+)- and (−)-RNA, respectively, of T36-CA identified seven nt variants at the extreme 5′ ends (Table 1). The group 1 consensus sequence (i.e. “AATTTCTCAA”) was found in 3 nt variants (Table 1, 5′ variants 1, 2, 3) from the Fillmore and Tulare samples. Remarkably, nt variants from both these samples also contained unique sequences similar, but not identical, to the group 2 consensus sequence (i.e. AATTTCA**C**AA) in that none of them contained the “**C**” at position 8 (Table 1, 5′ variants 4 to 7). “AATTTCTCAA” and “AATTTCAAA” will hereafter be referred to as the group 1 and 2 consensus sequences (specific to this study), respectively. Based on the DNA sequences derived from the (+)-RNA, the difference in abundance between the group 1 and 2 consensus sequences within the Fillmore is distinct from that seen in the Tulare sample – Fillmore sample: 25 group 1 (AATTTCTCAA) vs 27 group 2 (AATTTCAAA) consensus sequences out of 63 (39.7% and 42.9%, respectively) sequences analyzed; Tulare sample: 72 group 1 vs 12 group 2 consensus sequences out of 97 (74.2% and 12.4%, respectively) sequences analyzed (Table 1, 5′ variants 1 and 4). One extra nt (an adenylate [A] or a cytidylate [C]) was observed upstream of both groups of consensus 5′ end nt sequences. The extra “A” was seen in seven sequences (five from consensus group 1 and two from consensus group 2) among 63 (+)-DNA from the Fillmore sample, and in eight sequences (all from consensus group 1) among 97 (+)-DNA from the Tulare sample (Table 1, 5′ variants 2 and 5). The extra “C” was seen in four sequences (two each from the groups 1 and 2 consensus) from the Fillmore sample, and five sequences (all from consensus group 2) from the Tulare sample (Table 1, 5′ variants 3 and 6).

**Table 1:**
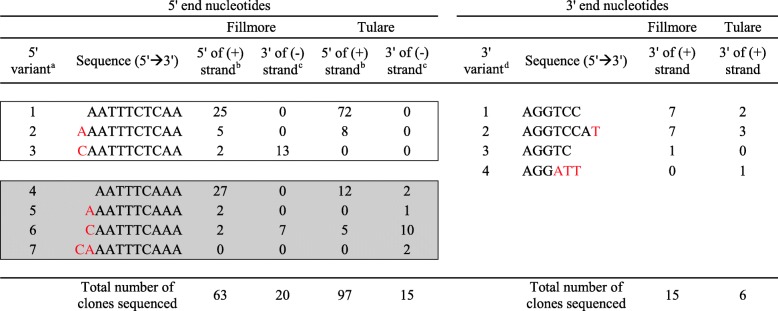
Nucleotide variations at the extreme termini in the genome of CTV with the T36-CA genotype

^a^The extreme 5′ end sequences of seven nt variants were identified by sequencing cloned DNA corresponding to the 5′ end of the T36-CA genome. In comparison with the T36 consensus genomic 5′ end nt sequences (black colored), the nt variants of T36-CA were found to contain extra/variant nt (s) (red colored nts). Group 1 nt variants (variants 1 to 3) (unshaded box) have consensus 5′ nt sequence “AATTTCTCAA” identical to that of T36 isolate FS674 (GenBank accession no. KC517485) and FS703 (GenBank accession no. KC517487). Group 2 nt variants (variants 4 to 7) (shaded box) have consensus 5′ nt sequence “AATTTCAAA” similar to that (“AATTTCACAA”) of the T36 sequences (GenBank accession nos. EU937521 and AY170468), except that it does not contain a “C” at position 8. The number of clones for each nt variant sequenced are as indicated

^b^The 5′ end nts of the T36-CA genome was determined by sequencing the (+)-DNA generated by 5′ RLM-RACE using the (+)-strand of dsRNA as a genetic template for reverse transcription (RT) and PCR amplification

^c^The 5′ end nts of the T36-CA genome was determined by 3′ RACE using the (−)-strand of dsRNA as a genetic template for reverse transcription (RT) and PCR amplification

^d^The extreme 3′ end sequences of 4 nt variants were identified by sequencing cloned (+)-DNA (generated by 3′ RACE) corresponding to the 3′ end of the T36-CA genome. The T36 consensus genomic 3′ end nts (black colored) and the T36-CA nt variants (red colored) are as shown. The number of clones for each nt variant sequenced are as indicated

Analysis of DNA sequences derived from the (−)-RNA indicated that an extra “C” was incorporated upstream of the first nt at the 5′ terminus of the (+)-RNA, as seen in 30 (20 from the Fillmore sample [consensus groups 1 and 2 combined] and 10 from the Tulare sample [all from consensus group 2]) out of 35 (85.7%) sequences examined (Table 1, 5′ variants 3 and 6). The (−)-RNA of the Tulare sample also contained a few other consensus group 2 variants – two sequences were identical to the consensus sequence (Table 1, 5′ variant 4), while another two contained a “CA” upstream of the consensus nts (Table 1, 5′ variant 7). Taken together, our results suggest that the consensus 5′ end nts of the (+)-RNA of T36-CA are likely “AAUUUC”, which are identical to those of other published sequences.

All full-length T36 genomic sequences from GenBank have a highly conserved “AGGTCCA” at their extreme 3′ ends. In this study, the (+)-DNA sequences derived from both the Fillmore and Tulare samples were consistent with “AGGTCCA” being the consensus – of the 21 sequences analyzed, nine contained “AGGTCC” (Table 1, 3′ variant 1). These sequences also contained an “A” (after the last “C”), which could not be distinguished from the first “A” of the poly (A) tail incorporated at the 3′ end of the RNA as part of the 3′ RACE procedure. However, in 10 of the 21 (47.6%) (+)-DNA sequences generated from the Fillmore and Tulare samples, an “A” was found after the “C” (Table 1, 3′ variant 2). These 3′ variant 2 sequences also contained an additional “T” before the poly (A) tract, indicating that “T” was the absolute terminal nt in these sequences. In other variants, deletion or replacement of nt (s) in the consensus “AGGTCCA” was observed. For example, the last two nts “C” and “A” was missing in 3′ variant 3; while in 3′ variant 4, “A”, “T” and “T” was found in place of “T”, “C” “C” and “A”. The frequency of these last two nt variants in the population is most likely low, as only one of each was present in the T36-CA (+)-DNA clones sequenced.

### Nucleotide heterogeneity at the genome ends of T30-CA

There are two consensus RNA sequences at the extreme 5′ end of multiple full-length T30 genomic sequences in GenBank – “AATTTCGATT” (e.g. GenBank accession nos. KC517489, KC517490, KC517491, and AF260651), and “ATTTTCGATT” (e.g. GenBank accession nos. Y18420, KC748391 and KU578007). Analysis of 99 (+)-DNA sequences corresponding to the 5′ end of the (+)-RNA of T30-CA (originating from Fillmore, CA) showed that only the consensus “AATTTCGATT” (Table 2, 5′ variant 1) was found in 94 (94.9%) of the sequences. Other less abundant nt variations were also observed – in 5′ variant 2, an additional “C” was seen upstream of the consensus “AATTTCGATT”, while in 5′ variant 3, the first consensus “A” was replaced with a “C” (Table 2). Analysis of the DNA sequences derived from the 3′ end of the (−)-RNA of T30-CA revealed that the most abundant 5′ end sequences (21 out of 22) were those of 5′ variant 2, while only 1 sequence belonged to that of 5′ variant 3 (Table 2).

**Table 2:**
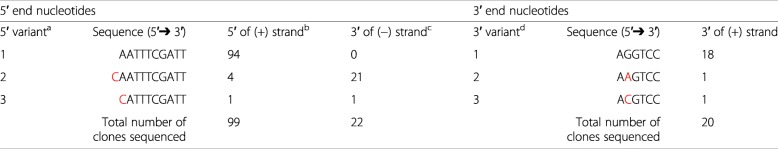
Nucleotide variations at the extreme termini in the genome of CTV with the T30-CA genotype

^a^The extreme 5′ end sequences of three nt variants were identified by sequencing cloned DNA corresponding to the 5′ end of the T30-CA genome. The consensus (black colored) nts located at the 5′ ends of other T30 sequences (GenBank accession nos. KC517489, KC517490, KC517491 and AF260651) were identified in the population of T30-CA in addition to nt variants with an extra nt (“C”, in red). The number of clones for each nt variant sequenced are as indicated

^b^The 5′ end nts of the T30-CA genome was determined by sequencing the (+)-DNA generated by 5′ RLM-RACE using the (+)-strand of dsRNA as a genetic template for reverse transcription (RT) and PCR amplification

^c^The 5′ end nts of the T30-CA genome was determined by sequencing the (−)-DNA generated by 3′ RACE using the (−)-strand of dsRNA as a genetic template for reverse transcription (RT) and PCR amplification

^d^The extreme 3′ end sequences of three nt variants were identified by sequencing cloned (+)-DNA (generated by 3′ RACE) corresponding to the 3′ end of the T30-CA genome. The conserved consensus nts and the variant nts are indicated in black and red, respectively. The number of clones for each nt variant sequenced are as indicated

All full-length T30 genomic sequences from GenBank have the consensus sequence “AGGTCCA” at their extreme 3′ ends. This was consistent with the results of sequencing 20 (+)-DNA clones corresponding to the 3′ end of the (+)-RNA of T30-CA. Most of the T30-CA sequences (18 out of 20) terminated with “AGGTCC” (Table 2, 3′ variant 1). Two other nt variants (3′ variants 2 and 3) were also identified but only one of each was found among the 20 clones sequenced (Table 2).

### The complete genome sequence of T36-CA

**Fig. 1:**
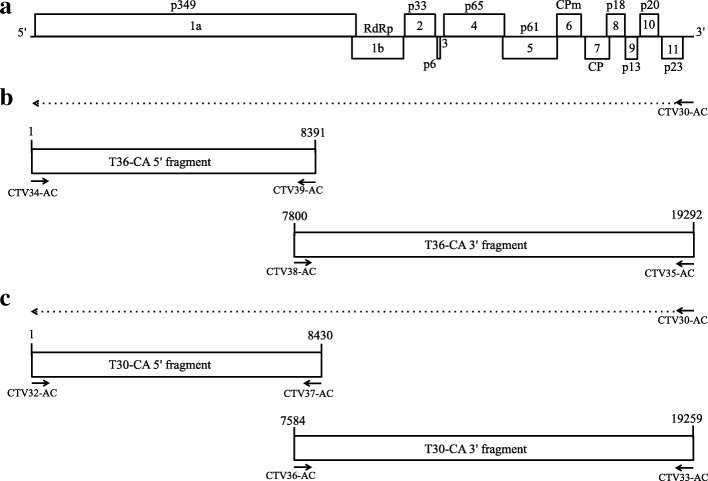
cDNA fragments corresponding to the genome of CTV with the T36-CA or the T30-CA genotype. **a** The genome organization of CTV. The numbered boxes (1a, 1b and 2 to 11) represent open reading frames (ORFs) and the proteins they encode (as indicated above or below the ORFs). RdRp, RNA-dependent RNA polymerase; CPm, minor coat protein; CP, major coat protein; and proteins named according to their predicted molecular weight, as indicated by the number (s) after the letter “p”. **b** To obtain the cDNA of the T36-CA genomic (g) RNA, the first strand cDNA (arrow with dotted line) was generated by reverse transcription (RT) using denatured T36-CA dsRNA and a reverse primer, CTV30-AC. The T36-CA 5′ fragment, corresponding to nucleotide (nt) position 1 to 8391, was PCR-amplified using primers CTV34-AC and CTV39-AC. The T36-CA 3′ fragment, corresponding to nt position 7800 to 19,292, was amplified using primers CTV38-AC and CTV35-AC. **c** The first strand cDNA of T30-CA (arrow with dotted line) was generated by the same method as described for T36-CA, using a reverse primer, CTV30-AC. Primers CTV32-AC and CTV37-AC were used to amplify the T30-CA 5′ fragment corresponding to nt position 1 to 8430, while primers CTV36-AC and CTV33-AC were used to amplify the T30-CA 3′ fragment (nt position 7584 to 19,259). Nucleotide sequences and other details of the primers (represented by solid arrows) used for generating the cDNA fragments are shown in Additional file 1: Table S1

Two overlapping cDNA fragments corresponding to the T36-CA genome were generated by RT-PCR using the T36-CA dsRNA (isolated from the Fillmore sample) and specific oligo primer sets (Additional file 1: Table S1), and sequenced. The two cDNA fragments, named T36-CA 5′ fragment and T36-CA 3′ fragment, contained the first 8391 nts and nts from position 7800 to 19,292, respectively, of the T36-CA genome (Fig. 1). Several of the cloned sequences were compared with that of a reference sequence from a Florida isolate of T36 (GenBank accession no. AY170468; here on after referred to as T36-FL) [30, 31]. Those that were most conserved compared with that of T36-FL, were selected and assembled to generate the full-length genomic sequence of T36-CA. No nt difference was found in the overlapped region (nt 7800–8391) between the selected 5′ and 3′ fragments, suggesting that both fragments were likely generated from the same nucleotide variant in the T36-CA population.

The genomic (g) RNA of T36-CA has 19,292 nts and shares a high nt identity (99.1%) with that of T36-FL (Table 3), differing by 178 nts. Among them, only one is a nt deletion i.e. the “C” at position 8 of the group 2 consensus nt sequence (Table 1) located at the 5′ UTR of T36-CA, while others are nt substitutions. The substituted nts are distributed along the entire T36-CA genome. Eight nt substitutions are in non-coding regions, including the 3′ UTR (one nt) and several intergenic regions (seven nts) (data not shown). The remaining nt substitutions are located within the open reading frames (ORFs). Overall, the nt identities of the UTRs and ORFs shared between T36-CA and T36-FL range from 98.1–99.7% (Table 3). The within-ORF nt substitutions would result in 68 deduced amino acid (aa) changes in T36-CA. These aa changes are present in 11 of the 12 ORFs and can be inferred by the < 100% (97.7 to 99.8%) aa identities between the corresponding ORFs of T36-CA and T36-FL (Table 3). The CPm coding sequence is the only T36-CA ORF that contains no aa difference compared to that of T36-FL.

**Table 3:** T36-CA nucleotide (nt) and deduced amino acid (aa) identities compared with other CTV strains

	T36-CA^a^		T36 -FL		T30-CA		SY568 (CA)		RB-AT25 (CA)		VT-AT39 (CA)	
Regions^b^	Number of nts	Number of aa	nt identity (%)	aa identity (%)	nt identity (%)	aa identity (%)	nt identity (%)	aa identity (%)	nt identity (%)	aa identity (%)	nt identity (%)	aa identity (%)
Full genome	19,292		99.1		81.7		81.5		90.8		80.8	
5′ UTR	106		99.1		55.3		58.1		99.1		60.6	
p349	9369	3123	99.1	98.9	72.9	74.5	73.2	74.9	90.6	74.5	73.2	75.1
RdRp	1503	501	99.6	99.8	80.0	92.0	77.1	89.5	90.5	96.0	81.1	90.9
p33	912	304	98.1	97.7	84.5	86.8	84.8	87.1	85.5	86.8	85.2	85.5
p6	156	52	98.7	98.0	89.7	96.1	89.7	96.1	88.5	96.1	91.0	96.1
p65 (HSP70h)	1785	595	99.3	99.2	94.1	96.5	94.1	96.6	88.4	94.3	89.2	95.0
p61	1608	536	99.0	98.1	95.4	96.6	96.7	96.8	94.2	95.9	88.6	91.8
p27 (CPm)	723	241	98.9	100.0	93.9	95.4	88.8	94.6	93.8	95.0	89.4	95.8
p25 (CP)	672	224	99.7	99.6	93.2	96.4	93.6	95.5	93.8	95.5	93.3	96.0
p18	504	168	99.4	99.4	95.2	95.8	92.7	94.0	96.2	95.2	58.4	95.2
p13	360	120	98.9	99.2	91.7	92.4	90.8	89.1	94.4	93.3	91.4	92.4
p20	549	183	99.1	97.8	88.5	92.3	91.4	96.2	90.5	93.4	90.7	94.5
p23	630	210	98.6	99.0	90.5	89.5	90.8	90.4	88.3	88.0	91.3	90.9
3′ UTR	273		99.6		97.4		97.8		97.1		97.1	

^a^A pairwise comparison was made between the genome sequence of T36-CA and that of the T36 genotype from Florida (T36-FL) or each of the sequences representing other CTV strains from California (CA), including T30-CA (obtained in this study), as indicated

^b^Regions in the CTV genome listed in the order of appearance from the 5′ to 3′ untranslated regions (UTR) s (as seen in Fig. 1)

Pairwise comparison of the T36-CA full-length nt sequence and those of CTV strains T30-CA, SY568, RB-AT25 and VT-AT39 show more variations (with nt identities ranging from 80.8 to 90.8%) than what is seen between T36-CA and T36-FL (99.1%) (Table 3 and Additional file 2: Figure S1). Pairwise comparison of the T36-CA nt sequence and those of T30-CA, SY-568 and VT-AT39 show relatively lower identities in the 5′ genomic region (the 5′ UTR, ORF1a and ORF 1b) than regions further downstream (p33 onwards) (Table 3). These results are consistent with previous reports suggesting that sequences at the 3′ proximal region of the genomes of CTV isolates are more conserved than those at the 5′ proximal region [5, 6]. In contrast, a high percentage nt identity is shared between T36-CA and RB-AT25 at their 5′ genomic regions. This is to be expected given that the latter is a recombinant strain with T36 sequences incorporated in the 5′ genomic region, including the 5′ UTR and part of ORF 1a [3, 26].

### The complete genome sequence of T30-CA

As with T36-CA, the complete genome sequence of T30-CA was determined by assembling the sequences from two cloned cDNA fragments generated by RT-PCR using T30-CA dsRNA (isolated from the Fillmore sample) and T30-specific oligo primers (Additional file 1: Table S1). The two cDNA fragments, named T30-CA 5′ fragment and T30-CA 3′ fragment, contained the first 8430 nts and nts from position 7584 to 19,259, respectively, of the T30-CA genome (Fig. 1). Both the 5′ and 3′ fragments were cloned, and multiple clones of each fragment were randomly selected and sequenced. The sequences were compared to that of T30-FL (GenBank accession no. AF260651) [5], a T30 genotype originating from Florida. The full-length genome of T30-CA was then assembled using the nts of the cloned 5′ and 3′ fragments that were most conserved compared to that of T30-FL.

T30-FL, T30-AT4 (the latter being a California strain of CTV with the T30 genotype isolated from *Citrus reticulata* Blanco [Murcott mandarin]) [4], and three other genotypes originating from CA – SY568, RB-AT25 and VT-AT39 – were used for the pairwise comparison of genomic sequences. T30-CA shares the highest nt sequence identity (99.4%) with both T30-AT4 and T30-FL compared to the other CTV genotypes (Table 4). The nt differences are distributed throughout the entire T30-CA genome (Table 4). In the non-coding regions (including 5′ and 3′ UTRs and intergenic regions), T30-CA differs from T30-AT4 and T30-FL by 3 and 5 nts, respectively. Among the 12 ORFs of T30-CA, 11 share nt identities, ranging from 99.1–99.8% and 98.4–99.7%, with the corresponding regions in T30-AT4 and T30-FL, respectively. Complete nt sequence identity (100%) was observed between ORF 3 (encoding p6) of T30-CA and that of both T30-AT4 and T30-FL (Table 4). A difference of 116 nts in the ORFs resulted in 52 deduced aa changes between T30-CA and T30-AT4, and the aa identities range from 98.4–100% between their corresponding ORFs (Table 4). When T30-CA was compared with T30-FL, a difference of 110 nts in the ORFs resulted in 51 deduced aa changes, with aa identities ranging from 96.7–100% between their corresponding ORFs (Table 4). In addition to ORF 3, ORF 10 (encoding p20) of both T30-AT4 and T30-FL, as well as ORF 9 (encoding p13) of T30-FL also share complete (100%) aa identity with the corresponding ORF (s) in T30-CA. The full-length T30-CA sequence share higher nt and aa identities with T30 sequences originating from different geographic regions (not shown) than with sequences of other CTV genotypes originating from California (Table 4 and Additional file 2: Figure S1). T30-CA shares the highest nt identity with SY568 among the other genotypes (T36-CA, RB-AT35 and VT-AT39) (Tables 3 and 4), consistent with previous findings that SY568 is a recombinant between T30 and other CTV stains [6].

**Table 4:** T30-CA nucleotide (nt) and deduced amino acid (aa) identities compared with other CTV strains

	T30-CA^a^		T30-AT4		T30-FL		SY568 (CA)		RB-AT25 (CA)		VT-AT39 (CA)	
Regions^b^	Number of nts	Number of aa	nt identity (%)	aa identity (%)	nt identity (%)	aa identity (%)	nt identity (%)	aa identity (%)	nt identity (%)	aa identity (%)	nt identity (%)	aa identity (%)
Full genome	19,259		99.4		99.4		93.1		82.4		90.3	
5′ UTR	108		99.1		97.2		73.1		55.8		75.7	
p349	9348	3116	99.4	99.2	99.5	99.4	90.8	92.0	72.8	99.2	90.9	92.3
RdRp	1434	478	99.7	99.6	99.4	99.4	91.6	96.4	84.0	94.8	91.3	97.0
p33	912	304	99.0	98.4	99.3	99.0	99.7	99.7	94.0	93.1	85.9	87.1
p6	156	52	100.0	100.0	100.0	100.0	100.0	100.0	96.8	100.0	93.6	96.1
p65 (HSP70h)	1785	595	99.3	98.8	99.6	99.0	99.8	99.5	88.2	94.1	88.7	94.6
p61	1608	536	99.2	98.9	99.1	98.7	99.2	99.1	95.4	96.3	88.2	90.8
p27 (CPm)	723	241	99.2	99.2	99.6	99.2	92.7	97.9	97.7	97.5	89.5	96.3
p25 (CP)	672	224	99.6	99.6	99.7	99.6	93.5	96.4	93.0	96.4	93.2	96.4
p18	504	168	99.4	99.4	99.2	98.2	93.1	95.8	94.8	94.6	58.0	97.0
p13	360	120	99.7	99.2	99.7	100.0	90.8	90.8	91.4	89.9	91.4	95.0
p20	549	183	99.8	100.0	99.5	100.0	93.3	95.1	93.3	95.6	91.6	93.4
p23	630	210	99.1	98.6	98.4	96.7	90.0	91.4	93.2	94.3	91.1	92.3
3′ UTR	274		99.6		99.3		97.8		97.8		98.5	

^a^A pairwise comparison was made between the genome sequence of T30-CA and those with the T30 genotype – T30-FL (a T30 genotype originating from Florida) and T30-AT4 (the only other California-based T30 genotype besides T30-CA to be completely sequenced), or each of the sequences representing other CTV strains from California (CA) as indicated

^b^Regions in the CTV genome listed in the order of appearance from the 5′ to 3′ untranslated regions (UTR) s (as seen in Fig. 1)

### Phylogenetic analysis

**Fig. 2:**
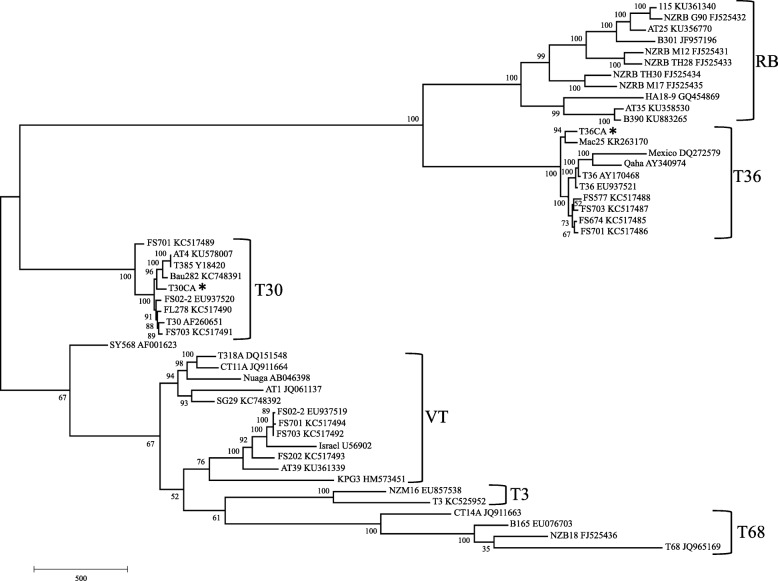
Phylogenetic relationships among six major genotypes of CTV based on their full-length genome sequences. The phylogenetic tree was constructed using the maximum parsimony method and bootstrap values (expressed as percentages of 1000 replicates) are indicated next to the branches. The names of isolate sequences and their accession numbers are shown at the tips of the branches. The T36-CA and T30-CA sequences determined in this study are both marked by an asterisk. RB, T36, T30, VT, T3 and T68 are the names of the six major genotypes. The branch lengths are scaled in units representing the number of nt changes over the entire genome sequence analyzed

To further characterize T36-CA and T30-CA, a phylogenetic tree was constructed using their full-length genome sequences and those of SY568 as well as 46 other isolates of six major CTV strains with the T36, T30, VT, RB, T3 and T68 genotypes [3]. Consistent with previous studies [3, 4, 26], T36-CA, T30-CA and CTV isolates belonging to the same genotypes were clustered together in the same clade within the phylogenetic tree (Fig. 2).

Within the T36 clade, T36-CA is clustered, with high bootstrap support, into the same linkage with Mac25 (GenBank accession no. KR263170) from Italy. The T36-CA nt sequence shared the highest nt identity (99.3%) with that of Mac25 among nine T36 sequences included in the phylogenic analysis. In contrast, T36-CA exhibited lower nt identities of 97.3% and 98.8% when compared with an isolate from Mexico (GenBank accession no. DQ272579) and isolate Qaha from Egypt (GenBank accession no. AY340974), respectively. The average nt identity between T36-CA and other sequences within the T36 clade was 98.8%.

Within the T30 clade, the nt sequences of both T30-CA and T30-AT4 are at the same sub-branch as the T30 from Europe, including T385 from Spain (GenBank accession no. Y18420) and Bau282 from Italy (GenBank accession no. KC748391). A pairwise comparison of the complete nt sequences showed 98.9–99.5% identities (on average 99.4%) between T30-CA and other T30 strains from Spain, Italy and US (i.e. GenBank accession nos. KU578007, AF260651, EU937520, KC517489, KC517491 and KC517490). The lowest nt identity (98.9%) was between T30-CA and FS701 (GenBank accession no. KC517489).

## Discussion

This study has identified the predominant consensus nts and the nt heterogeneity at the extreme 5′ and 3′ termini of two prevalent CTV genotypes, T36 and T30, from California, as well as the nt variants present in the genomes of these viruses. The T36-CA infected samples harbored two predominant groups of 5′ terminal consensus nts, such as those exemplified by 5′ variants 1 and 4 (Table 1) i.e. “AATTTCTCAA” and the unique “AATTTCAAA”, respectively, that are identical to (e.g. isolate FS674 [KC517485] and isolate FS703 [KC517487]), or similar with (e.g. isolate FS02–2 [EU937521] and T36-FL [AY170468]) those of other CTV isolates with the T36 genotype. In contrast, the T30-CA infected samples contained only one predominant set of 5′ terminal consensus nts (5′ variant 1) (Table 2) that is identical to one of two sets of T30 consensus nts in GenBank. This is consistent with a previous report showing a high degree of nt conservation of T30 strains from different geographic regions separated by more than a hundred years [5]. Some of the nt variants of T36-CA and T30-CA were found to contain an extra “C” upstream of the consensus nts (5′ variants 3 and 6 [Table 1] and 5′ variant 2 [Table 2]). The presence of an unpaired “G” at the extreme 3′ end of the (−)-RNA of the CTV dsRNA (i.e. with no complementary “C” on the [+]-RNA) has been observed previously, and this is postulated to be a common feature for the alphavirus-like superfamily of viruses, to which CTV belongs [1, 17]. This feature also was seen in T36-CA and T30-CA when we analyzed the (−)-DNA sequences (Tables 1 and 2). Interestingly, an extra “C” was found in the (+)-DNA sequences of T36-CA and T30-CA, suggesting that the (+)-strand of CTV dsRNA, with an extra “C” incorporated upstream of the consensus nts, exists in the sequence population. It is unclear whether the “C” on the 5′ end of the (+)-RNA serves as a template for the “G” at the 3′ of the (−)-RNA during replication.

Nucleotide variations at the extreme 3′ end of the CTV genomic RNA are less compared to those located at the extreme 5′ end, whether among or within CTV genotypes. For example, many CTV genomic sequences in GenBank have the highly conserved “AGGTCCA” at their 3′ ends. In this study, most of the 3′ (+)-DNA clones of both T36-CA and T30-CA sequenced were found to end with “AGGTCC” (Tables 1 and 2). Because the 3′ RACE procedure incorporates a poly “A” tail immediately downstream of the final “C”, any “As” that might exist downstream of it were indistinguishable from those of the poly “A” tail. Taking this caveat into consideration, it is likely that “AGGUCCA” are the predominant consensus nts at the 3′ termini of the (+)-RNA of both T36-CA and T30-CA. It might have been possible to determine the 3′ end nts of the genomic RNA by performing RLM-RACE using the (−)-RNA. However, in our hands, this procedure was unsuccessful in yielding any cDNA products corresponding to the 5′ end of the (−)-RNA. Another nt variant (AGGTCCAT) at the 3′ ends of T36-CA (3′ variant 2) (Table 1) also was observed in many sequences and this suggested that an additional “U” could be incorporated downstream of the consensus 3′ end ribonucleotides. For CTV, several studies reported that nt variants with non-template nts incorporated downstream of the 3′ end consensus nts in the (+)-RNA were identified when the DNA products generated from viral dsRNA were sequenced [1, 17, 32]. However, there were disparities in these reports. For example, in one study, a non-template “U” was identified downstream of the last “CCA” in the (+)-RNA of a FL strain of T36 [1]. In another study, the T36 genome was found to end with “CC” and it was suggested that an “A” might be incorporated downstream of “CC” in the (+)-RNA as a non-template nt [32]. Our results suggested that the T36-CA population contains both “CCA” and “CCAU” at the extreme 3′ end of the (+)-RNA (3′ variants 1 and 2) (Table 1). The “CCA” has been shown to be important for virus replication [14] but the additional “U” after the “A” appears to be neither required for virus replication nor systematic infection [13, 30, 31].

The determination of nts at the extreme ends of the CTV genome by sequencing cDNA clones derived from viral dsRNA was previously done for other T36 and T30 isolates obtained from various sources and locations outside California, including Florida, USA and Spain [1, 5, 6, 17, 18]. However, none of these studies included any comprehensive analysis on the nt heterogeneity at the extreme ends of the CTV genomes. Our study contrasts with that of Lopez et al. [17] and others [1, 5, 6, 18] in two regards. First, the nt variations seen in our results are specific to California strains of T36 and T30, and little or no information on nt heterogeneity in the genome ends is available for CTV of any genotypes. Furthermore, one of the predominant sets of T36-CA 5′ end consensus (AATTTCAAA) is unique and this has never been documented. Second, the information of the extreme 5′ end nts were derived from the (+)- and the (−)-RNA using 5′ RLM-RACE and 3′ RACE, respectively, thus allowing us to more thoroughly analyze the nts in the regions being queried. In contrast, polyadenylated (+)- or (−)-RNA of the viral dsRNA was used to determine the sequence information of the genome ends of CTV in all of the studies reported in the literature [1, 5, 6, 13, 17, 18]. Our sequencing results clearly identified the nt heterogeneity at the extreme 5′ end of T36-CA (e.g. 5′ variants 2, 4, 5 and 7 [Table 1]) that has not been reported before for other isolates with the T36 genotype, and this information would have likely been missed had polyadenylated (+)- or the (−)-RNA alone been used.

The above findings have clearly provided useful guidelines for strategies to clone the predominant CTV sequences within their respective populations. For example, using an oligo primer containing the nts of 5′ variant 4 (Table 1), we were able to incorporate the unique consensus “AATTTCAAA” into the RT-PCR amplified T36-CA 5′ cDNA fragment. In addition, identification of the consensus nts (AAUUUCGAUU) at the 5′ end of T30-CA RNA population also allowed us to design the appropriate oligo primers to incorporate these nts into the T30-CA 5′ cDNA fragment. Knowledge of the additional 5′ and 3′ end nts or nt substitutions seen in some of the variants would give us the option of incorporating them into the full-length infectious cDNA clones of T36-CA and T30-CA in the future. These infectious clones can be used to investigate whether the nt variants at the extreme ends of the CTV genome are involved in any biological functions.

Recently, the complete genome sequence of T30-AT4, a CTV with the T30 genotype originating from Fillmore, CA, was determined using small (s) RNA deep sequencing [4]. Here, our analyses have shown that the T30-CA genome shares 99.4% identity with that of T30-AT4. Although the T30-CA isolate also originated from Fillmore, CA, it was isolated from *Citrus aurantifolia* (Mexican lime), while T30-AT4 was isolated from *Citrus reticulata* Blanco (Murcott mandarin). This suggests that the host species may have some influence on the distribution of the major nt variant sequences in a CTV population [33]. However, whether or not the two different host species could influence the nt heterogeneity at the extreme ends of the T30-CA and T30-AT4 genomes remains unknown since information on nt heterogeneity at the terminal ends of T30-AT4 is not available.

Pairwise comparisons of the complete genome sequences of T36-CA or T30-CA with those of other isolates from the same genotypes (Tables 3 and 4; data not shown) consistently showed a high degree of genetic conservation (> 97.3% nt identity for T36 and > 98.9% nt identity for T30), between the sequences [3, 5]. In contrast, the nt identity between T36-CA and T30-CA is only 81.7%, and both sequences also show higher nt diversity compared to the reference sequences of the different genotypes (e.g. SY568, RB-AT25 and VT-AT39) found in California (Tables 3 and 4). Collectively, these results are consistent with our knowledge of the genotypic diversity within CTV. The determination of the complete genome sequences of the two genotypes, T36-CA and T30-CA, prevalent in California will pave the way for ongoing studies aimed at engineering a CTV-based vector suitable for specific molecular biotechnology applications.

## Conclusions

Extensive sequence analysis of the extreme ends of the T36-CA and T30-CA genomes has revealed the presence of novel as well as conserved nt variants. The terminal nt information has been essential in facilitating the cloning of the full-length T36-CA and T30-CA genomes for the determination of their complete nt sequences and phylogenetic relationships with CTV isolates of the same and different genotypes. The data generated from this study will add to the growing resource of information on the complex genetic diversity and geographic distribution of CTV.

## Additional files

Additional file 1: **Table S1.** Oligonucleotide primers used for the 5′ RLM-RACE-, 3′ RACE- and RT-/PCR-mediated amplification of CTV genomic RNA. (DOCX 26 kb)

Additional file 2: **Figure S1.** The color coded matrix of pairwise nucleotide identity between two full-length CTV sequences. The complete sequences of California T36-CA and T30-CA were used in the pairwise comparison with those of a T36 (T36-FL) and a T30 (T30-FL) genotype from Florida, respectively, as well as with representative sequences of different CTV genotypes (SY468, VT-AT39 and RB-AT25) originating from California. The colors represent the degree of identity between two sequences as indicated in the color scale. (PDF 267 kb)

## Abbreviations

Aa: Amino acid
CA: California
CTV: *Citrus tristeza virus*
FL: Florida
Nt: Nucleotide
ORF: Open reading frame
RLM-RACE: RNA ligase-mediated rapid amplification end of cDNA end
UTR: Untranslated region

## References

[1] Karasev AV, Boyko VP, Gowda S, Nikolaeva OV, Hilf ME, Koonin EV (1995). Complete sequence of the *Citrus tristeza virus* RNA genome. Virology.

[2] Karasev AV (2000). Genetic diversity and evolution of closteroviruses. Annu Rev Phytopathol.

[3] Harper SJ (2013). *Citrus tristeza virus*: evolution of complex and varied genotypic groups. Front Microbiol.

[4] Yokomi Raymond, Selvaraj Vijayanandraj, Maheshwari Yogita, Chiumenti Michela, Saponari Maria, Giampetruzzi Annalisa, Weng Ziming, Xiong Zhongguo, Hajeri Subhas (2018). Molecular and biological characterization of a novel mild strain of citrus tristeza virus in California. Archives of Virology.

[5] Albiach-Martí MR, Mawassi M, Gowda S, Satyanarayana T, Hilf ME, Shanker S (2000). Sequences of *Citrus tristeza virus* separated in time and space are essentially identical. J Virol.

[6] Vives MC, Rubio L, López C, Navas-Castillo J, Albiach-Martí MR, Dawson WO (1999). The complete genome sequence of the major component of a mild *Citrus tristeza virus* isolate. J Gen Virol.

[7] Drake JW, Holland JJ (1999). Mutation rates among RNA viruses. Proc Natl Acad Sci U S A.

[8] García-Arenal F, Fraile A, Malpica JM (2001). Variability and genetic structure of plant virus populations. Annu Rev Phytopathol.

[9] Kong P, Rubio L, Polek M, Falk BW (2000). Population structure and genetic diversity within California *Citrus tristeza virus* (CTV) isolates. Virus Genes.

[10] Rubio L, Ayllón MA, Kong P, Fernández A, Polek M, Guerri J (2001). Genetic variation of *Citrus tristeza virus* isolates from California and Spain: evidence for mixed infections and recombination. J Virol.

[11] Albiach-Martí MR, Robertson C, Gowda S, Tatineni S, Belliure B, Garnsey SM (2010). The pathogenicity determinant of *Citrus tristeza virus* causing the seedling yellows syndrome maps at the 3′-terminal region of the viral genome. Mol Plant Pathol.

[12] Gowda S, Satyanarayana T, Ayllón MA, Moreno P, Flores R, Dawson WO (2003). The conserved structures of the 5′ nontranslated region of *Citrus tristeza virus* are involved in replication and virion assembly. Virology.

[13] Satyanarayana T, Gowda S, Boyko VP, Albiach-Martí MR, Mawassi M, Navas-Castillo J (1999). An engineered closterovirus RNA replicon and analysis of heterologous terminal sequences for replication. Proc Natl Acad Sci U S A.

[14] Satyanarayana T, Gowda S, Ayllón MA, Albiach-Martí MR, Dawson WO (2002). Mutational analysis of the replication signals in the 3′-nontranslated region of *Citrus tristeza virus*. Virology.

[15] Satyanarayana T, Gowda S, Ayllón MA, Dawson WO (2004). Closterovirus bipolar virion: evidence for initiation of assembly by minor coat protein and its restriction to the genomic RNA 5′ region. Proc Natl Acad Sci U S A.

[16] Simon AE, Miller WA (2013). 3′ cap-independent translation enhancers of plant viruses. Annu Rev Microbiol.

[17] López C, Ayllón MA, Navas-Castillo J, Guerri J, Moreno P, Flores R (1998). Molecular variability of the 5′- and 3′-terminal regions of citrus tristeza virus RNA. Phytopathology.

[18] Ayllón MA, López C, Navas-Castillo J, Garnsey SM, Guerri J, Flores R, Moreno P (2001). Polymorphism of the 5′ terminal region of *Citrus tristeza virus* (CTV) RNA: incidence of three sequence types in isolates of different origin and pathogenicity. Arch Virol.

[19] Yokomi RK, Saponari M, Sieburth PJ (2010). Rapid differentiation and identification of potential severe strains of *Citrus tristeza virus* by real-time reverse transcription-polymerase chain reaction assays. Phytopathology.

[20] Salem NM, Chen AYS, Tzanetakis IE, Mongkolsiriwattana C, Ng JCK (2009). Further complexity of the genus *Crinivirus* revealed by the complete genome sequence of *Lettuce chlorosis virus* (LCV) and the similar temporal expression of LCV genomic RNAs 1 and 2. Virology.

[21] Valverde RA, Nameth ST, Jordan RL (1990). Analysis of double stranded RNA for plant virus diagnosis. Plant Dis.

[22] Martin RR, Jelkmann W, Tzanetakis IE, Hadidi A, Barba M, Candresse T, Jelkmann W (2011). Double-stranded RNAs and their use for characterization of recalcitrant viruses. Virus and virus-like diseases of pome and stone fruits.

[23] Chen AYS, Pavitrin A, Ng JCK (2012). Agroinoculation of the cloned infectious cDNAs of *Lettuce chlorosis virus* results in systemic plant infection and production of whitefly transmissible virions. Virus Res.

[24] Muhire Brejnev Muhizi, Varsani Arvind, Martin Darren Patrick (2014). SDT: A Virus Classification Tool Based on Pairwise Sequence Alignment and Identity Calculation. PLoS ONE.

[25] Yang ZN, Mathews DM, Dodds JA, Mirkov TE (1999). Molecular characterization of an isolate of *Citrus tristeza virus* that causes severe symptoms in sweet orange. Virus Genes.

[26] Yokomi RK, Selvaraj V, Maheshwari Y, Saponari M, Giampetruzzi A, Chiumenti M (2017). Identification and characterization of *Citrus tristeza virus* isolates breaking resistance in trifoliate orange in California. Phytopathology.

[27] Kumar S, Stecher G, Tamura K (2016). MEGA7: molecular evolutionary genetics analysis version 7.0 for bigger datasets. Mol Biol Evol.

[28] Nei M, Kumar S (2000). Molecular evolution and phylogenetics.

[29] Felsenstein J (1985). Confidence limits on phylogenies: an approach using the bootstrap. Evolution.

[30] Ambrós S, El-Mohtar C, Ruiz-Ruiz S, Peña L, Guerri J, Dawson WO (2011). Agroinoculation of *Citrus tristeza virus* causes systemic infection and symptoms in the presumed nonhost *Nicotiana benthamiana*. Mol Plant-Microbe Interact.

[31] El-Mohtar C, Dawson WO (2014). Exploring the limits of vector construction based on *Citrus tristeza virus*. Virology.

[32] Karasev AV, Nikolaeva OV, Mushegian AR, Lee RF, Dawson WO (1996). Organization of the 3′-terminal half of beet yellow stunt virus genome and implications for the evolution of closteroviruses. Virology.

[33] Ayllón MA, Rubio L, Sentandreu V, Moya A, Guerri J, Moreno P (2006). Variations in two gene sequences of *Citrus tristeza virus* after host passage. Virus Genes.

