# Supplementary material for: Nucleotide heterogeneity at the terminal ends of the genomes of two California Citrus tristeza virus strains and their complete genome sequence analysis
Source: Virol J. 2018 Sep 15;15:141. doi: 10.1186/s12985-018-1041-4 (PMC6139129; doi:10.1186/s12985-018-1041-4)
Supplement: Supplementary file 1 — Table S1. Oligonucleotide primers used for the 5′ RLM-RACE-, 3′ RACE- and RT-/PCR-mediated amplification of CTV genomic RNA. (DOCX 26 kb) [file 12985_2018_1041_MOESM1_ESM.docx]

Additional file 1 **Table S1** Oligonucleotide primers used for the 5' RLM-RACE-, 3' RACE- and RT-/PCR-mediated amplification of CTV genomic RNA**.**

| **Primer**  **(specificity)^1^** | **Polarity^2^** | **Sequence (5'🡪3')** | **Primer Position^3^** | **Amplification** | **Usage/ Remarks** |
| --- | --- | --- | --- | --- | --- |
| CTV349-AC  (T36) | - | CCGTTGCAGGCATGGTTCCAAAAGT | 794-770 | 5' RLM-RACE**^4^** | RT and the first PCR step to determine the 5' end nts |
| CTV350-AC  (T36) | - | AGAAGCGAGAAGAGGCCCGGCTAGA | 542-518 | 5' RLM-RACE | Nested PCR step to determine the 5' end nts |
| CTV18-SW  (T36) | - | CTTACCAAGTCGGCTGTTTCGTCGG | 664-640 | 3' RACE^5^ | First PCR step to determine the 5' end nts |
| CTV24-SW  (T36) | - | GCAGAGGTGAGCGGAGGTTTG | 471-451 | 3' RACE | Nested PCR step to determine the 5' end nts |
| CTV21-SW  (T36) | + | GGATTGCGGTAGAAAACACGATAAGG | 18596-18621 | 3' RACE | First PCR step to determine the 3' end nts |
| CTV22-SW  (T36) | + | ATGCGTTCTCCGGAAGAAACTCC | 18849-18871 | 3' RACE | Nested PCR to determine the 3' end nts |
| CTV30-AC  (T36; T30) | - | TGGACCTATGTTGGCCCCCCATAG | 19292-19269 (T36-CA)  19259-19236 (T30-CA) | RT-PCR | Anneals to the extreme 3' terminal 24 nts; generates the first strand cDNA of the full-length genomic RNA |
| CTV34-AC  (T36) | + | GACTA*GGGCCC*AATTTCAAAATTCAACCTGTTCGCCCAG | 1-28 | PCR | Anneals to the extreme 5' terminal 28 nts; generates the 5' cDNA fragment; designed with an ApaI site (italicized) |
| CTV35-AC  (T36) | - | CTAAG*CTCGAG*TGGACCTATGTTGGCCCCCCATAG | 19292-19269 | PCR | Anneals to the extreme 3' terminal 24 nts; generates the 3' cDNA fragment; designed with a XhoI site (italicized) |
| CTV38-AC  (T36) | + | GACTA*GGGCCC*CAGTGCCTGACATGTACCCTGTTGC | 7800-7824 | PCR | Pairs with CTV35-AC to generate the 3' cDNA fragment; designed with an ApaI site (italicized) |
| CTV39-AC  (T36) | - | CTAAG*CTCGAG*GCTAGCTTCCTGCCCATCAATTCATTAG | 8391-8364 | PCR | Pairs with CTV34-AC to generate the 5' cDNA fragment; designed with a XhoI site (italicized) |
| CTV345-AC  (T30) | - | GGTTGGCAGCGGAAGACACGTCATT | 925-901 | 5' RLM-RACE | RT and the first PCR step to determine the 5' end nts |
| CTV346-AC  (T30) | - | GCCCGGCTGAGAAAGAATGCAGAAT | 530-506 | 5' RLM-RACE | Nested PCR step to determine the 5' end nts |
| CTV2-SW  (T30) | - | ACAGTAGGGTCAACTAGTTTCGCAAC | 614-589 | 3' RACE | First PCR step to determine the 5' end nts |
| CTV3-SW  (T30) | - | CGAACAGAGAACGGAAAACAG | 311-291 | 3' RACE | Nested PCR step to determine the 5' end nts |
| CTV6-SW  (T30) | + | TGCGTGGATTGTGGTAGAAA | 18557-18576 | 3' RACE | First PCR step to determine the 3' end nts |
| CTV7-SW  (T30) | + | CGGTATGACTGCTAAGGCAAT | 18853-18873 | 3' RACE | Nested PCR step to determine the 3' end nts |
| CTV32-AC  (T30) | + | GCCCA*ATGCAT*AATTTCGATTCAAATTCACCCGTACCTCC | 1-29 | PCR | Anneals to the extreme 5' terminal 29 nts; generates the 5' cDNA fragment; designed with a NsiI site (italicized) |
| CTV33-AC  (T30) | - | CTAAG*ATGCAT*TGGACCTATGTTGGCCCCCCATAG | 19259-19236 | PCR | Anneals to the extreme 3' terminal 24 nts; generates the 3' cDNA fragment; designed with a NsiI site (italicized) |
| CTV36-AC  (T30) | + | GCCCA*ATGCAT*GGCGAACGGTAGCGTTGTATCTGA | 7584-7607 | PCR | Pairs with CTV33-AC to generate the 3' end cDNA fragment; designed with a NsiI site (italicized) |
| CTV37-AC  (T30) | - | CTAAG*ATGCAT*CATCAAATAAGAGTCCACGGTGAACACTCTACCG | 8430-8397 | PCR | Pairs with CTV32-AC to generate the 5' end cDNA fragment; designed with a NsiI site (italicized) |

^1^The genomic RNA of CTV, with genotype indicated in parenthesis, to which the oligo primer anneals.

^2^A (+) polarity oligo primer anneals to the (-)-RNA. A (-) polarity oligo primer anneals to the (+)-RNA.

^3^Oligo primers for 5' RNA ligase-mediated rapid amplification of cDNA ends **(**RLM-RACE) and 3' RACE are specific to conserved nucleotides (nt)s in the 5' and 3' regions of selected accessions of CTV with the T36 (GenBank KC517485, KC517487, and AY170468) and T30 (GenBank KC517490, KC517491, AF260651) genotypes. The primer positions indicated are based on the nt positions in the T36-CA or T30-CA genomes.

^4^Decapped denatured double stranded (ds)RNA was ligated to the GeneRacer RNA oligo (5'-CGACUGGAGCACGAGGACACUGACAUGGACUGAAGGAGUAGAAA-3') and subjected to reverse transcription (RT) using the genotype-specific primer, CTV349-AC or CTV345-AC, followed by two separate PCR steps (i.e. first PCR and nested PCR). Oligo primer pairs used for the first PCR were, in the forward direction: GeneRacer 5' primer (5'-CGACTGGAGCACGAGGACACTGA-3') and the strain-specific primer, CTV349-AC or CTV345-AC. Oligo primer pairs used for nested PCR were GeneRacer 5' nested primer (5'-GGACACTGACATGGACTGAAGGAGTA-3') and the strain-specific primer, CTV350-AC or CTV346-AC.

^5^Denatured dsRNA was subjected to “A” tailing of the terminal nt, followed by RT using the oligo primer AAP(A), 5'-GGCCACGCGTCGACTAGTACTTTTTTTTTTTTTTTT-3', and two separate PCR steps (i.e. first PCR and nested PCR). Oligo primer pairs used for the first PCR were the strain-specific primer, CTV18-SW, CTV21-SW, CTV2-SW or CTV6-SW and the AUAP primer (5'-GGCCACGCGTCGACTAGTAC-3'). Oligo primer pairs used for the nested PCR were the AUAP primer and the strain-specific primer, CTV24-SW, CTV22-SW, CTV3-SW or CTV7-SW.
