# Supplementary figures and images for: Nucleotide heterogeneity at the terminal ends of the genomes of two California Citrus tristeza virus strains and their complete genome sequence analysis
Source: Virol J. 2018 Sep 15;15:141. doi: 10.1186/s12985-018-1041-4 (PMC6139129; doi:10.1186/s12985-018-1041-4)

Figure S1

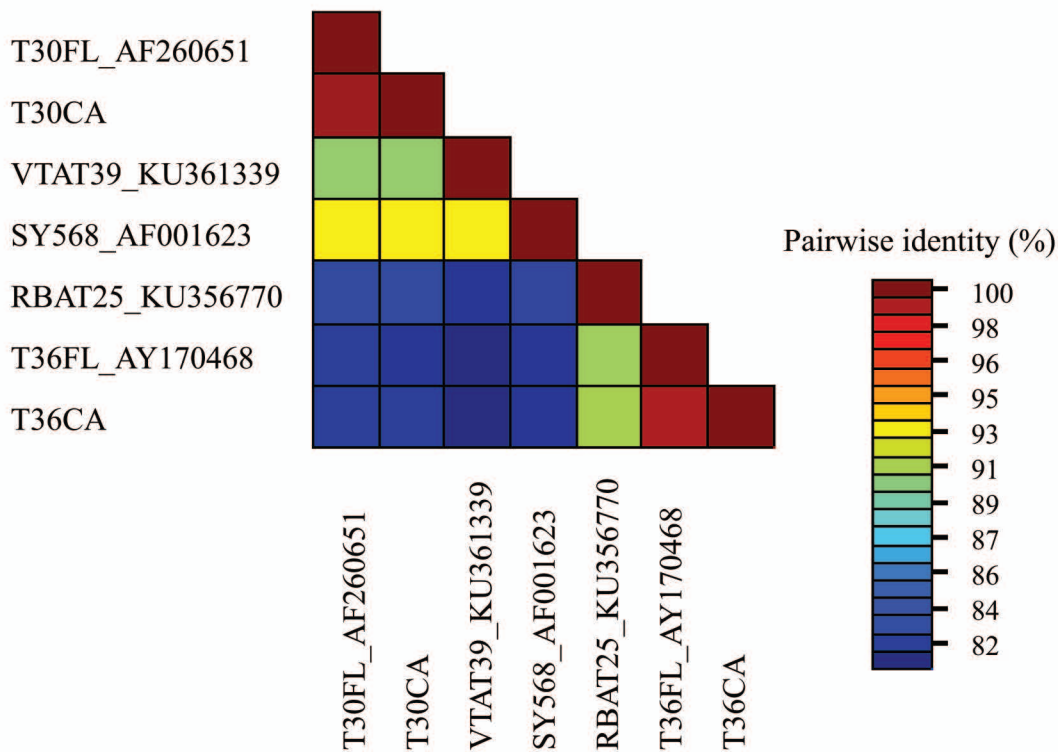

Supplement: Supplementary file 2 — Figure S1. The color coded matrix of pairwise nucleotide identity between two full-length CTV sequences. The complete sequences of California T36-CA and T30-CA were used in the pairwise comparison with those of a T36 (T36-FL) and a T30 (T30-FL) genotype from Florida, respectively, as well as with representative sequences of different CTV genotypes (SY468, VT-AT39 and RB-AT25) originating from California. The colors represent the degree of identity between two sequences as indicated in the color scale. (PDF 267 kb) [file 12985_2018_1041_MOESM2_ESM.pdf]
